# Phillygenin protects against lipopolysaccharide-induced acute lung injury by acting as a PXR agonist via NF-κB inhibition

**DOI:** 10.1038/s41598-026-66771-z

**Published:** 2026-08-29

**Authors:** Cong-jie Wang, Meng-da Dai, Yu-jun Tan, Chang-jun Lv, Yan Song, Sheng-ping Zhai, Zhe Chen

**Affiliations:** 1 Department of Respiratory and Critical Care Medicine, Tianjin Medical University General Hospital, Tianjin, 300052 China; 2 Department of Respiratory and Critical Care Medicine, Yantai Yuhuangding Hospital, Yantai, 264000 China; 3 Department of Pharmacy, Shengli Oilfield Central Hospital, Dongying, 257034 China; 4 College of Pharmacy, Inner Mongolia Medical University, Hohhot, 010110 China; 5 Department of Respiratory and Critical Care Medicine, Shandong Medical and Pharmaceutical University Hospital, Binzhou, 256600 China; 6 Department of Geriatrics, Tianjin Geriatrics Institute, Tianjin Medical University General Hospital, Tianjin, 300052 China; 7 Department of General Practice, Yantai Yuhuangding Hospital, Yantai, 264000 China

**Keywords:** Phillygenin, Pregnane X receptor, NF-κB, RXRα, Acute lung injury, Biochemistry, Cell biology, Diseases, Drug discovery, Medical research

## Abstract

Acute lung injury (ALI) and acute respiratory distress syndrome (ARDS) are life-threatening conditions with substantial mortality and limited targeted therapies. The pregnane X receptor (PXR) has emerged as a promising therapeutic target for inflammatory diseases, though its role in ALI remains underexplored. This study characterizes phillygenin (PHI), a bioactive lignan isolated from Forsythia suspensa, as a PXR agonist, elucidating its protective mechanism in lipopolysaccharide (LPS)-induced ALI. PHI binding to PXR was characterized by ligand fishing using hollow fibers and luciferase reporter assays, revealing direct agonistic activity. In LPS-stimulated human bronchial epithelial cells, PHI-activated PXR competed with NF-κB p65 for RXRα, suppressing NF-κB p65 nuclear translocation and downstream inflammatory cascades. Concurrently, PHI upregulated antioxidant enzymes (GPX-2/GPX-7) and anti-apoptotic proteins (Bcl-2/Bcl-xL), while downregulating pro-inflammatory mediators (TNF-α, IL-6), in a PXR-dependent manner. PXR knockdown abolished these protective effects, confirming target specificity. In vivo, PHI dose-dependently attenuated lung histopathological injury, reduced inflammatory cytokine levels in bronchoalveolar lavage fluid, and mitigated alveolar epithelial apoptosis in LPS-challenged mice. These findings support PHI as a direct PXR agonist and the PHI-PXR-NF-κB axis as a mechanistic foundation for developing targeted interventions in acute inflammatory lung diseases.

**Supplementary Information:**

The online version contains supplementary material available at https://doi.org/10.1038/s41598-026-66771-z.

## Introduction

Acute lung injury (ALI) can advance to a more serious condition known as acute respiratory distress syndrome (ARDS)^1^. Collectively, they embody a range of life-threatening pulmonary disorders. They are characterized by widespread damage to the alveolar epithelium and vascular endothelium, leading to uncontrolled inflammation and impaired gas exchange^2,3^. The global burden of Acute Respiratory Distress Syndrome (ARDS) is profound, with an incidence of approximately 10% in intensive care units and associated in-hospital mortality nearing 40%^4^. This high mortality rate is consistently observed across different regions, as evidenced by specific cohort studies, and is recognized as a persistent clinical challenge in current medical guidelines^5,6^. Furthermore, survivors frequently experience persistent complications, including cognitive impairment, physical disability, pulmonary deficiency, and neuromuscular weakness, affecting their quality of life^7^. Patients and social healthcare systems are seriously affected by these problems^4–7^. In such a challenging situation, the lack of targeted treatment methods underscores the urgent need for effective intervention strategies.

The pathophysiological mechanisms of ALI mainly involve uncontrolled inflammatory responses and apoptosis of alveolar epithelial cells (AECs)^2,8^. As the key targets of injury, AECs play a crucial role in ALI progression. Moreover, damage to alveolar epithelial cells (AECs) is an initial sign of ALI/ARDS^8,9^. Lipopolysaccharide (LPS), a key pathogenic factor derived from Gram-negative bacteria, can trigger the core pathological processes of ALI by disrupting immune and inflammatory homeostasis^10,11^. The main mechanism of action of LPS relies on the NF-κB activation, which is a critical regulatory molecule driving downstream inflammatory cascades^12^. NF-κB activation leads to the excessive release of key pro-inflammatory cytokines such as TNF-α, IL-6, and IL-1β^13,14^. Elevated TNF-α, IL-1β, IL-6, IL-8, and IL-18 signal poor prognosis, and track closely with morbidity and mortality^15^. Additionally, NF-κB activation upregulates inducible nitric oxide synthase (iNOS) expression and stimulates reactive oxygen species (ROS) production, leading to oxidative stress and subsequent cellular degeneration^16,17^. The uncontrolled inflammatory response and persistent oxidative stress act synergistically, exacerbating lung tissue damage and directly promoting alveolar epithelial cell apoptosis. This interplay serves as a core driver in the progression of ALI^3,8^.

The pregnane X receptor (PXR), a ligand-activated nuclear receptor, functions primarily as a transcriptional regulator of downstream gene expression^18^. The canonical role of PXR in xeno- and endobiotic metabolism—mediated via RXRα heterodimerization to induce enzymes like CYP3A4—is well-established^19,20^. Its involvement in inflammatory signaling has recently emerged as an important regulatory mechanism. Numerous studies indicate that PXR activation exerts significant anti-inflammatory effects, potentially through direct inhibition of NF-κB p65 and negative regulation of its downstream targets^18,21^. This interaction between nuclear receptor signaling pathways and the innate immune system further highlights the important value of PXR as a potential therapeutic target for inflammation-related diseases^22,23^. In recent years, studies have directly demonstrated that targeted activation of PXR produces clear therapeutic benefits in models of inflammatory bowel disease and related conditions^24^. Despite this mechanistic insight, PXR-targeted therapy for ALI remains underdeveloped, with its NF-κB-modulatory mechanism incompletely defined, offering a significant translational opportunity.

Phillygenin (PHI), a well-characterized lignan isolated from *Forsythia suspensa* (Thunb.), is widely used in clinical practice for its heat-clearing and detoxifying properties^25,26^. Preclinical studies indicate that PHI exhibits potent anti-inflammatory and antioxidant properties, demonstrating good pharmacological potential^27,28^. Moreover, its application as an active ingredient in approved clinical preparations further highlights its translational medical value^27,29^. However, a key scientific question remains unanswered: previous studies have suggested that certain plant-derived compounds have the potential to activate PXR^30,31^, and it has been hypothesized that PHI may exert its effects through a similar mechanism. Currently, there is insufficient direct evidence to show that PHI provides a protective effect against ALI/ARDS. It is also unclear whether its potential mechanism depends on the activation of PXR. Addressing this issue is of high importance for the advancement of this field.

In summary, this study tests the core hypothesis that PHI protects against LPS-induced ALI through PXR-dependent NF-κB inhibition. Specifically, it identified PHI as a direct PXR agonist and characterized its binding properties. Furthermore, it clarified the mechanism whereby PHI-activated PXR competitively disrupts NF-κB nuclear translocation via RXRα heterodimerization. In addition, it examined PXR-dependent cytoprotective, antioxidant, and anti-apoptotic effects in cellular and murine ALI models. Ultimately, it established the PHI-PXR-NF-κB axis as a translational intervention strategy for ALI/ARDS therapy.

## Results

### PHI mitigates apoptosis and inflammation in LPS-challenged bronchial epithelial cells

**Fig. 1:**
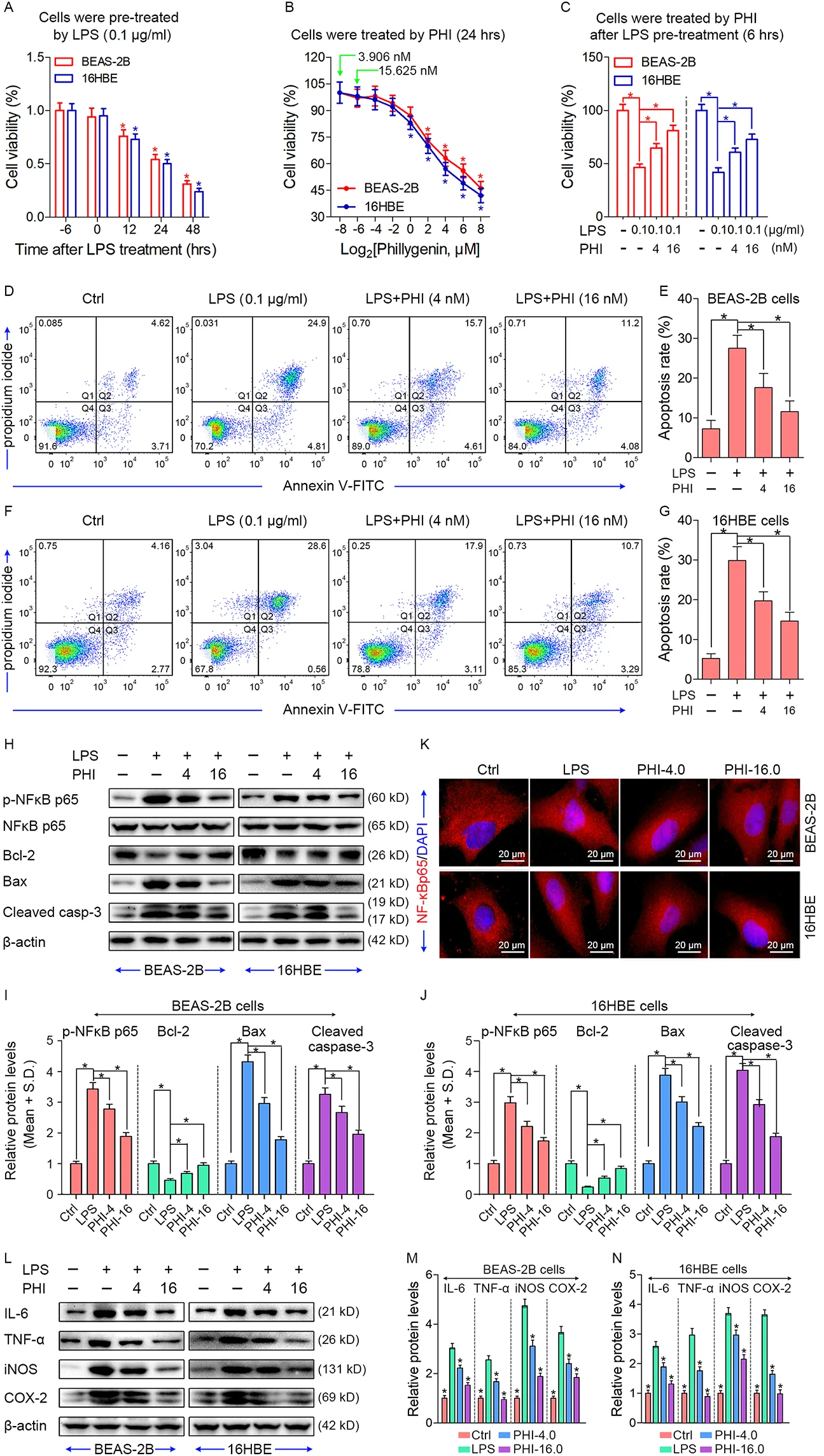
PHI suppresses LPS-induced apoptosis in airway epithelial cells by targeting NF-κB signaling. After pre-treatment with LPS (1.0 µg/mL) for 6 h, BEAS-2B and 16HBE cells were treated with or without PHI. Cell viability (**A**–**C**) and apoptosis (**D**–**G**) were detected by CCK-8 and flow cytometry assay. The protein expressions of NF-κB p65 (phosphorylated and non-phosphorylated), Bcl‑2, Bax, cleaved caspase‑3 were detected by immunoblot analysis (**H**–**J**). Immunocytochemistry for NF‑κB p65 nuclear translocation (**K**). The expression of IL-6, TNF-α, iNOS, COX-2 was detected (**L**–**N**). Data are presented as mean ± S.D. (*n* = 3). *P < 0.05. Full-length, uncropped blots are presented in Supplementary Figure S2.

To evaluate the protective potential of PHI against LPS‑induced cytotoxicity, we first established an in vitro injury model. LPS (1.0 µg/mL) pre-treatment for 6 h reduced the viability of BEAS‑2B and 16HBE cells by approximately 50% following 24 h of LPS-free culture (Fig. 1A). PHI alone suppressed viability in a concentration‑dependent manner, with minimal cytotoxicity at 4 nM and 16 nM (Fig. 1B). However, when combined with LPS pre‑treatment for 6 h, PHI at 4 and 16 nM reversed the LPS‑induced decline in viability and suppressed apoptosis in both BEAS‑2B and 16HBE cells (Fig. 1C-G). These findings suggest that PHI safeguards bronchial epithelial cells from LPS-induced cytotoxicity.

NF-κB mediates LPS-induced cell death and inflammation. To investigate whether PHI suppresses NF-κB to exert its protective effects, we assessed the expression and subcellular localization of NF-κB. Its nuclear translocation is a definitive marker of pathway activation. Specifically, the detection of phosphorylated NF-κB signifies the activation of this key transcription factor—a well-established hallmark of the lung injury response^8,13^. PHI prevented LPS-induced NF‑κB p65 phosphorylation and nuclear translocation, without changing the total NF-кB p65 protein level (Fig. 1H–K). This LPS-induced activation upregulated pro‑apoptotic proteins (Bax, cleaved caspase‑3) while downregulating the anti‑apoptotic protein Bcl‑2 (Fig. 1H–J). Furthermore, NF‑κB activation enhanced the expression of inflammatory mediators, including IL‑6, TNF‑α, iNOS, and COX‑2, in both BEAS‑2B and 16HBE cells (Fig. 1L–N). Accordingly, PHI treatment restored the balance of apoptosis‑related proteins, including Bax, Bcl‑2, and cleaved caspase‑3. It also significantly reduced the inflammatory response in both BEAS‑2B and 16HBE cells (Fig. 1L–N). Collectively, our findings show that PHI mediates protection against LPS-induced damage in bronchial epithelial cells by coordinately suppressing NF-κB signaling and its downstream apoptotic and inflammatory pathways.

### PHI directly binds to PXR and promotes PXR-RXRα heterodimerization

**Fig. 2:**
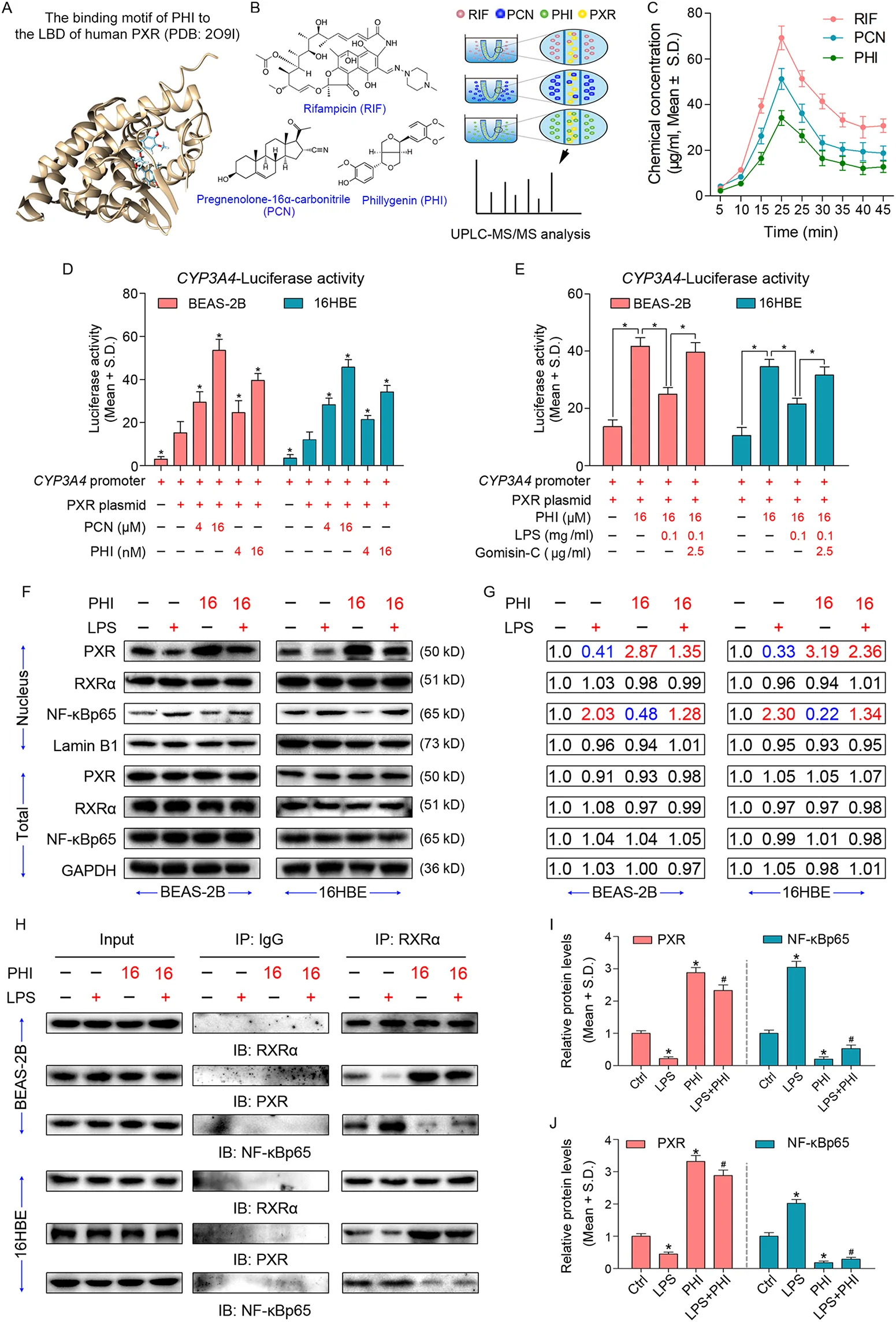
PHI blocks NF-κB nuclear translocation by binding to PXR. The predicted binding mode of PHI to human and mouse PXR from molecular docking (**A**). The chemical structures and binding kinetics of PHI, PCN and RIF to PXR (**B**,**C**). PXR activation by PHI and PCN in BEAS-2B and 16HBE cells (**D**,**E**). Expression of PXR, RXRα, and NF‑κB p65 was analyzed by immunoblotting (**F**,**G**). Co-immunoprecipitation assays demonstrated the binding capacity of PHI-activated PXR and NF-κB p65 to RXRα (**H**–**J**). Data are presented as mean ± S.D. (*n* = 3). *P < 0.05 vs. control; #*P* < 0.05 vs. LPS group. Full-length, uncropped blots are presented in Supplementary Figure S2.

To identify the molecular target of PHI, bioinformatic analysis via BATMAN‑TCM (http://bionet.ncpsb.org.cn/batman-tcm/) predicted PXR as a potential binding partner (Fig. 2A). To examine whether PHI could bind to PXR, we utilized hollow fibers containing PXR protein to ‘capture’ the ligand molecules, with PCN (a mouse PXR activator) and RIF (a human PXR activator) as positive controls. The dissociated ligands (PHI, RIF and PCN) were identified and quantified by LC-MS. The chemical structures of PHI, RIF and PCN, alongside a schematic of the screening workflow, are presented in Fig. 2B. As expected, PHI and PXR showed a clear binding phenomenon, and the binding kinetics curve reached its peak at 25 min (Fig. 2C). This suggests that PHI could interact with PXR, although with weaker binding capacity than that of RIF and PCN. Moreover, considering the species specificity of PXR, the hollow fiber-based ligand fishing (HFLF) strategy not only confirmed the binding of PHI to human PXR as described above, but also demonstrated its binding to mouse PXR (Fig. S1 A and B). To further evaluate the effects of PHI on PXR activity, BEAS‑2B and 16HBE cells were co‑transfected with the dual-luciferase reporter plasmid pGL3‑CYP3A4 (containing CYP3A4 promoter, whose expression was highly sensitive to PXR) and pcDNA3.1‑PXR, followed by treatment with or without PHI/PCN/RIF. Both PHI and PCN enhanced PXR‑driven activation of the CYP3A4 promoter (Fig. 2D), confirming that PHI activates mouse PXR. Furthermore, the promoting ability of human PXR on CYP3A4 promoter was also enhanced by RIF and PHI (Fig. S1B), suggesting that PHI is also an activator of human PXR. However, LPS partially attenuated PHI-induced PXR activation. This attenuation was reversed by the NF‑κB inhibitor Gomisin‑C (Fig. 2E), indicating that LPS‑induced NF‑κB p65 activation suppresses PXR activity.

To further investigate the relationship between PXR and NF-κB p65, western blot analysis revealed that LPS promoted NF-κB p65 nuclear translocation while inhibiting PXR nuclear translocation in both BEAS-2B and 16HBE cells. In contrast, PHI promoted PXR but suppressed NF-κB p65 nuclear translocation (Fig. 2F, G). Notably, the total protein levels of PXR, NF-κB p65, and RXRα remained unchanged upon treatment with either LPS or PHI. Moreover, neither ligand altered the expression or nuclear localization of RXRα, which is known to mediate the nuclear translocation of both PXR and NF-κB p65 through heterodimerization^32,33,34^. The next question is whether PHI inhibits the nuclear translocation of NF-κB p65 by enhancing the binding ability of PXR to RXRα. Co‑immunoprecipitation (Co‑IP) revealed distinct ligand‑dependent interaction patterns: LPS enhanced the association between RXRα and NF‑κB p65, whereas PHI promoted RXRα‑PXR heterodimer formation. Notably, under PHI exposure, RXRα showed stronger binding to PXR than to NF‑κB p65, suggesting that PHI may competitively shift RXRα binding from NF‑κB p65 toward PXR, thereby potentially inhibiting NF‑κB p65 nuclear translocation and activity via RXRα sequestration (Fig. 2H-J). Results indicated that PHI, a direct PXR agonist, reversed LPS-activated NF-κB p65 by promoting PXR-RXRα assembly. This competitive shift in RXRα binding inhibited NF‑κB nuclear translocation and activation, and finally, the effect of LPS was diminished.

### PXR activation enhances cellular antioxidant defenses and suppresses apoptosis

**Fig. 3:**
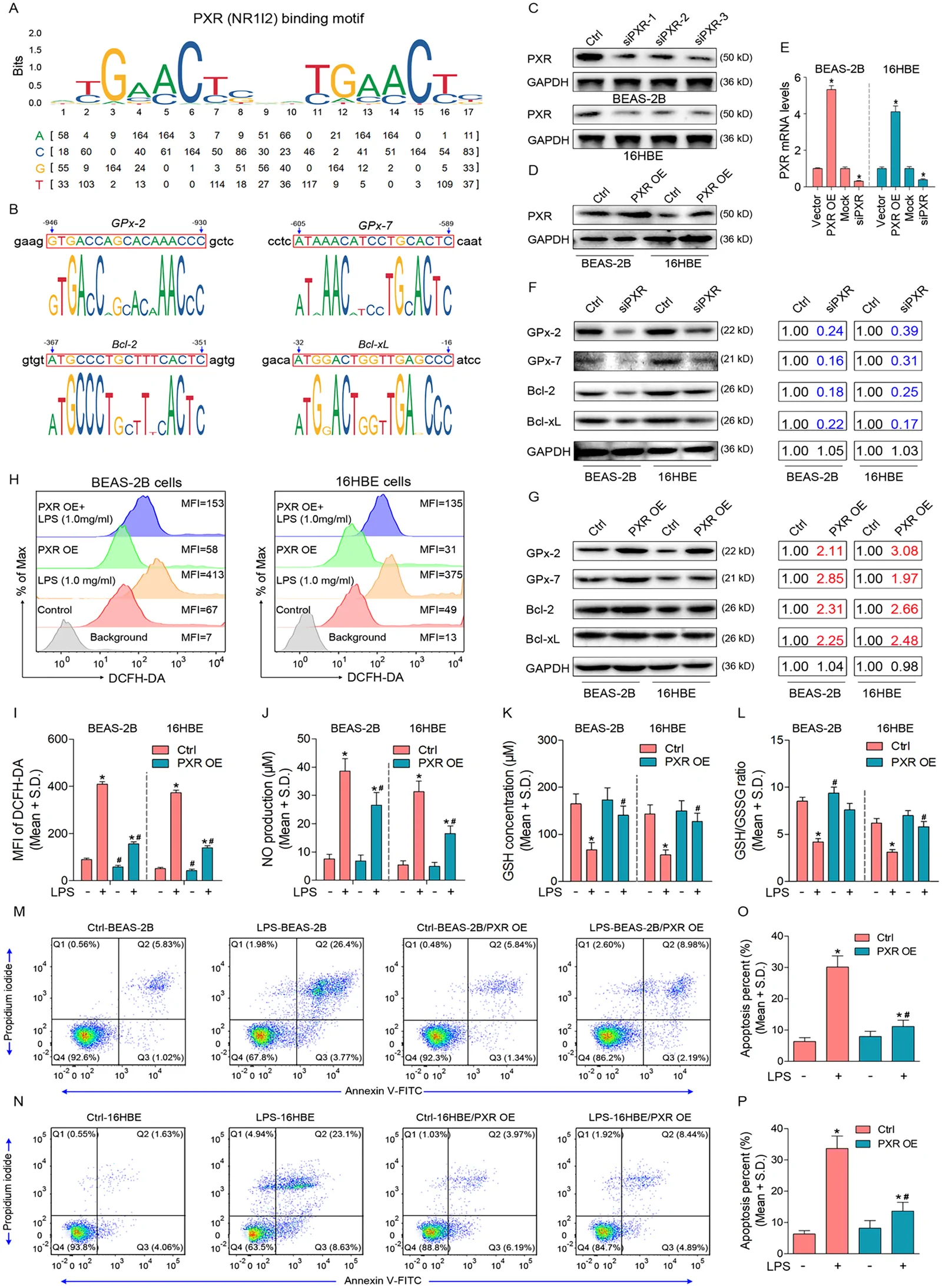
PXR activation attenuates LPS-induced apoptosis by enhancing antioxidant defenses and upregulating anti-apoptotic proteins. Putative PXR binding sites in the promoter regions of GPX-2, GPX-7, Bcl-2, and Bcl-xL were predicted using the JASPAR database (**A**,**B**). Validation of PXR knockdown (siPXR) and overexpression (PXR-OE) efficiency in BEAS-2B and 16HBE cells (**C**–**E**). Effects of PXR knockdown and overexpression on GPX-2, GPX-7, Bcl‑2, and Bcl‑xL protein expression (**F**–**G**). The role of PXR in modulating LPS-induced oxidative stress was assessed by measuring intracellular ROS (**H**,**I**), NO (**J**), GSH levels (**K**), and the GSH/GSSG ratio (**L**). The anti-apoptotic effect of PXR was evaluated by flow cytometry in BEAS-2B (**M**,**O**) and 16HBE (**N**,**P**) cells. Data are presented as mean ± S.D. (*n* = 3). *P < 0.05 vs. control; ^#^*P* < 0.05 vs. LPS group. Full-length, uncropped blots are presented in Supplementary Figure S2.

Building on the established PHI-PXR interaction, we further explored PXR’s cytoprotective functions. To address this, potential antioxidant and anti‑apoptotic target genes of PXR were identified through a binding motif, which predicted PXR‑binding sites in the promoters of GPX-2, GPX-7, Bcl‑2, and Bcl‑xL (Fig. 3A,B). To functionally validate this regulation, we generated PXR‑silenced (siPXR) and PXR‑overexpressing (PXR OE) models in both BEAS-2B and 16HBE cells. Western blot and qPCR confirmed efficient PXR knockdown and overexpression (Fig. 3C–E), enabling downstream functional analysis. The outcomes suggested that GPX-2, GPX-7, Bcl-2, and Bcl-xL levels were downregulated by siPXR but increased by PXR-OE in both BEAS-2B and 16HBE cells (Fig. 3F,G). As key glutathione peroxidases, GPX-2 and GPX-7 function to eliminate damaging ROS such as hydrogen peroxide, thereby protecting the structural and functional integrity of cellular membranes^35^. Excessive ROS accumulation is a known trigger of apoptosis^36^. Since GPX-2, GPX-7, and iNOS are all critically involved in ROS metabolism^37^, we hypothesized that PXR might suppress apoptosis by modulating cellular ROS levels. Consistent with this, PXR overexpression significantly attenuated the LPS‑induced elevation in total ROS (Fig. 3H,I). Specifically, it reduced the overproduction of nitric oxide (NO, a key reactive nitrogen species) (Fig. 3J), and concurrently increased the level of the antioxidant glutathione (GSH) and the GSH/GSSG ratio (Fig. 3K,L). These findings indicated that PXR activation enhances the cellular antioxidant defense system, leading to a reduction in oxidative stress. Consistent with the observed attenuation of oxidative stress, PXR upregulation significantly suppressed LPS‑induced apoptosis (Fig. 3M–P). Mechanistically, PXR activation simultaneously enhanced antioxidant defenses by elevating GPX-2 and GPX-7 expression, increasing the GSH/GSSG ratio, and reducing NO overproduction, thereby counteracting oxidative stress‑mediated apoptotic signaling. This coordinated response was further supported by the upregulation of the anti‑apoptotic proteins Bcl‑2 and Bcl‑xL (Figs. 3F, G). Collectively, PXR activation orchestrated a coordinated cytoprotective response that integrates antioxidant and anti‑apoptotic pathways, ultimately alleviating LPS‑induced cytotoxicity in bronchial epithelial cells.

### The protective effects of PHI are PXR-dependent

**Fig. 4:**
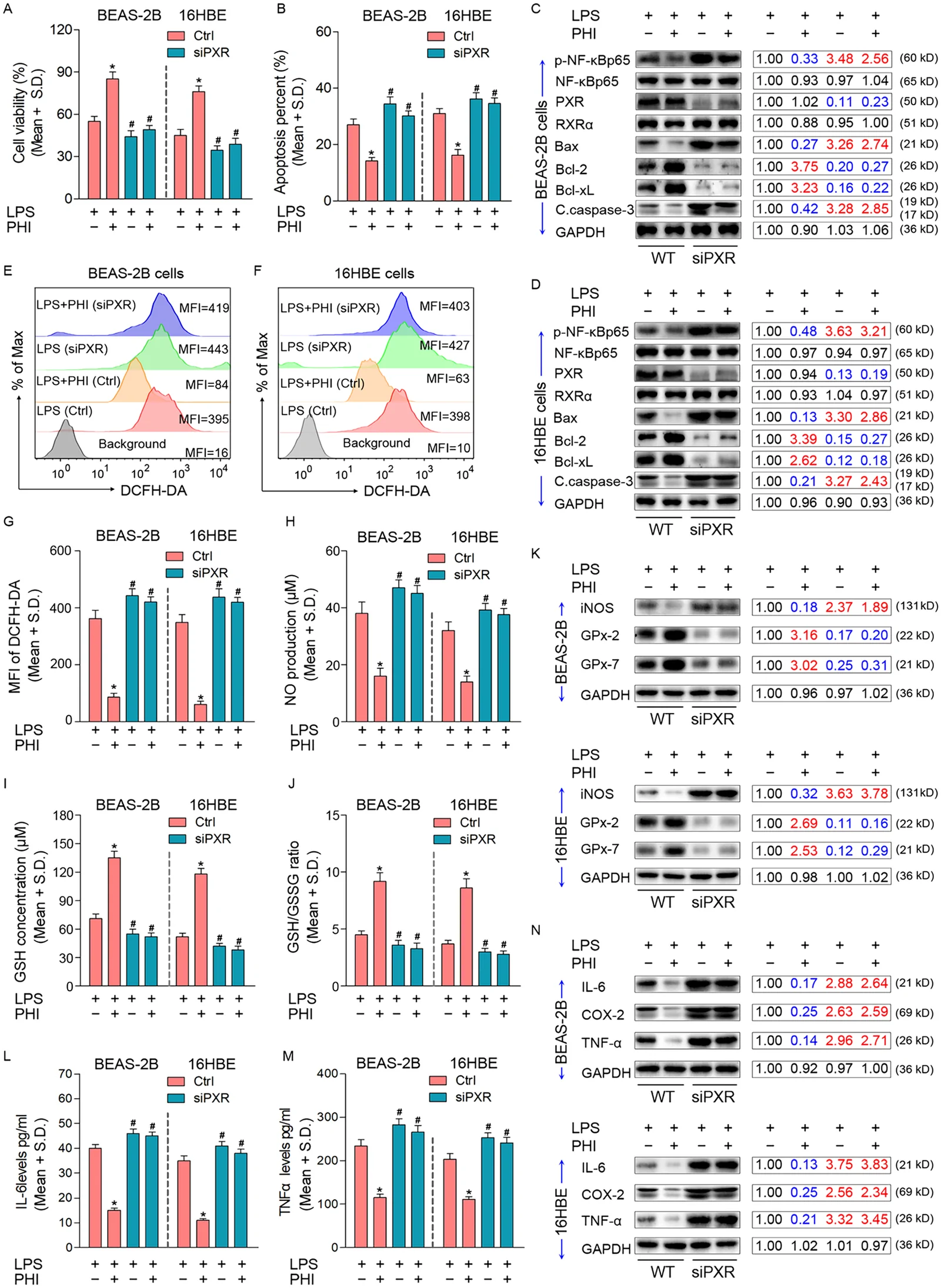
The cytoprotective effects of PHI against LPS are mediated by PXR. PXR knockdown reversed the protective effects of PHI on LPS-impaired cell viability and apoptosis (**A**,**B**). The apoptosis‑regulating proteins (Bax, Bcl‑2, Bcl‑xL, cleaved caspase‑3), NF-κB p65 (total and phosphorylated), PXR, and RXRα expressions (**C**,**D**). The antioxidative capacity of PHI, evidenced by reduced ROS, NO, GSH and GSH/GSSG ratio, was also abolished upon PXR silencing (**E**–**J**). Similarly, PXR was necessary for PHI to regulate iNOS, GPX-2, GPX-7, IL‑6, COX‑2, and TNF‑α (**K**–**N**). Data are presented as mean ± S.D. (*n* = 3). *P < 0.05 vs. control; #*P* < 0.05 vs. LPS + PHI group. Full-length, uncropped blots are presented in Supplementary Figure S2.

To investigate the contribution of PXR to PHI-induced therapeutic outcomes, this study performed loss‑of‑function experiments using PXR knockdown. When PXR was silenced, PHI no longer rescued the LPS‑induced loss of cell viability (Fig. 4A) and its anti‑apoptotic effect was abolished (Fig. 4B). Following PXR knockdown, the protective efficacy of PHI against LPS‑induced damage was markedly attenuated. This was evidenced by its diminished ability to suppress NF‑κB activity, lower pro‑apoptotic protein (Bax, cleaved caspase‑3) expression, or maintain anti‑apoptotic protein (Bcl‑2, Bcl‑xL) levels (Fig. 4C,D). The above results indicate that PHI depends on PXR to control the expression of several important proteins. We further examined whether PXR is required for PHI’s regulation of oxidative stress. In PXR‑deficient cells, PHI did not attenuate LPS‑induced ROS accumulation (Fig. 4E–G) and lost its ability to lower NO or restore the GSH/GSSG ratio (Fig. 4H–J). Accordingly, PHI could no longer suppress iNOS upregulation or restore GPX-2/GPX-7 expression (Fig. 4K). Building on previous evidence that PHI suppresses LPS‑induced inflammatory cytokine expression, we further investigated whether this effect is mediated through PXR. The results indicated that although PHI significantly suppressed LPS‑induced expression of IL‑6, TNF‑α, and COX‑2, this inhibitory effect was abolished upon PXR knockdown in both cell models (Fig. 4L–N). In conclusion, PHI‑induced PXR activation underlies its anti‑inflammatory effects. In summary, our findings identify PXR as a critical molecular target in LPS‑induced epithelial injury. PXR-dependent processes are mainly used by PHI to provide its fundamental protective properties.

### PHI alleviates acute lung injury in LPS‑induced mice

**Fig. 5:**
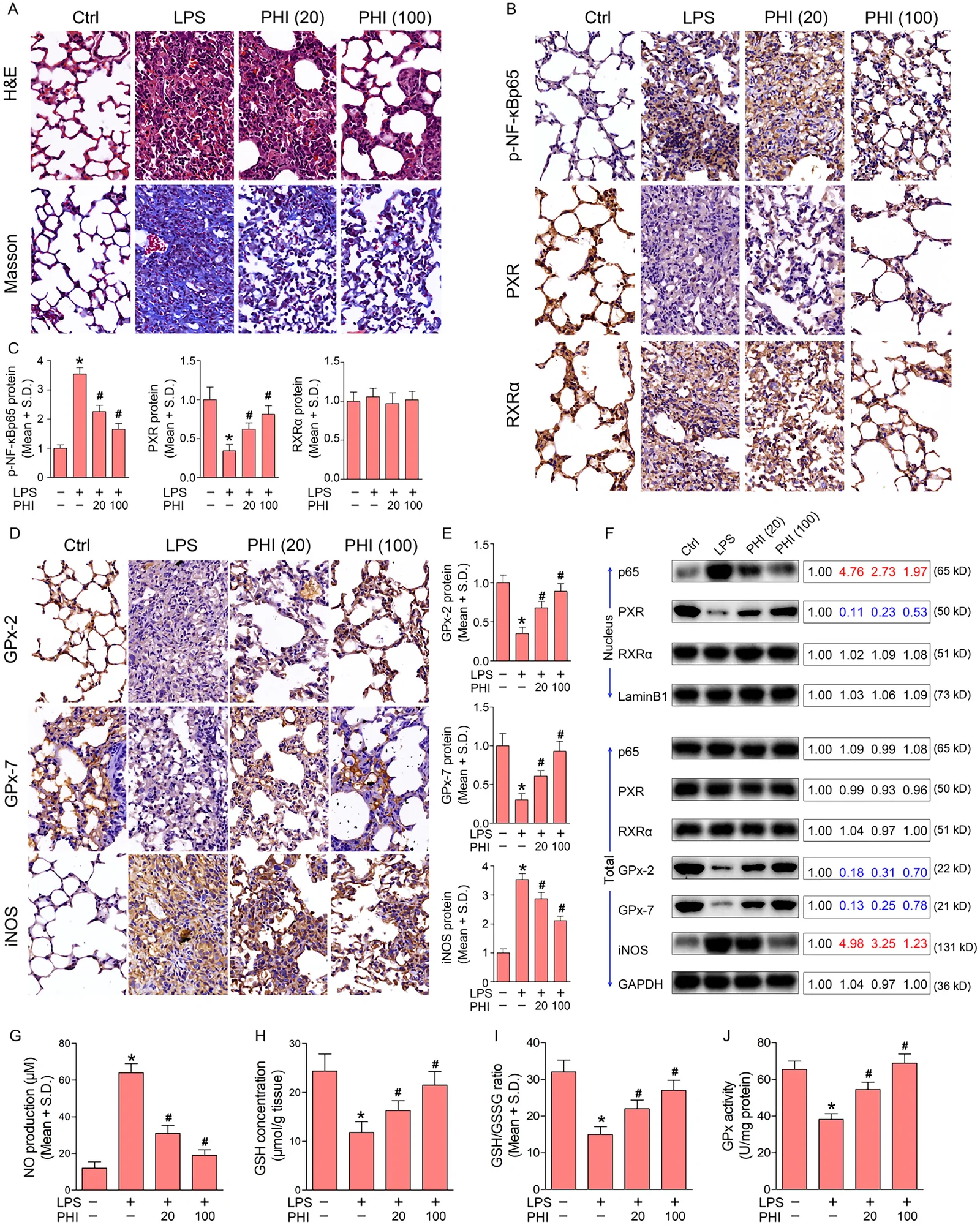
PHI improves acute lung injury induced by LPS in vivo. Lung histopathology in an LPS‑induced mouse model was evaluated using H&E and Masson staining (**A**). Protein levels of NF-κB p65, PXR, RXRα, GPX-2, GPX-7, and iNOS were evaluated by immunohistochemistry (**B**–**E**) and western blotting (**F**). Biochemical analysis of lung homogenates included measurements of NO production (**G**), GSH concentration (**H**), the GSH/GSSG ratio (**I**), and GPx activity (**J**). Data are presented as mean ± S.D. (*n* = 3). *P < 0.05 vs. control; #*P* < 0.05 vs. LPS group. Original magnification: 40× (**A**,**B**,**D**). Full-length, uncropped blots are presented in Supplementary Figure S2.

To validate the cytoprotective role of PHI in vivo, we established an LPS‑induced acute lung injury model in C57BL/6 mice. Histological analysis was performed using H&E and Masson staining. PHI concentration‑dependently attenuated LPS‑induced lung tissue injury (Fig. 5A). IHC analysis showed that LPS promoted phosphorylation of NF-κB p65 and suppressed PXR expression, while PHI treatment could reverse the above effects. In addition, RXRα expression did not differ significantly among the groups (Fig. 5B,C). Since p‑NF‑κB p65 upregulates iNOS^38,39^, and PXR enhances GPX‑2 and GPX‑7 expression^40^, these downstream markers showed corresponding changes: PHI treatment increased GPX-2 and GPX-7 while reducing iNOS levels, consistent with Western blot data (Fig. 5D–F). Furthermore, PHI treatment resulted in reduced NO, increased GSH levels and GSH/GSSG ratios, as well as enhanced GPX activity (Fig. 5G–J). These data indicate that PHI reduces oxidative stress and helps restore redox balance in the injured lung.

**Fig. 6:**
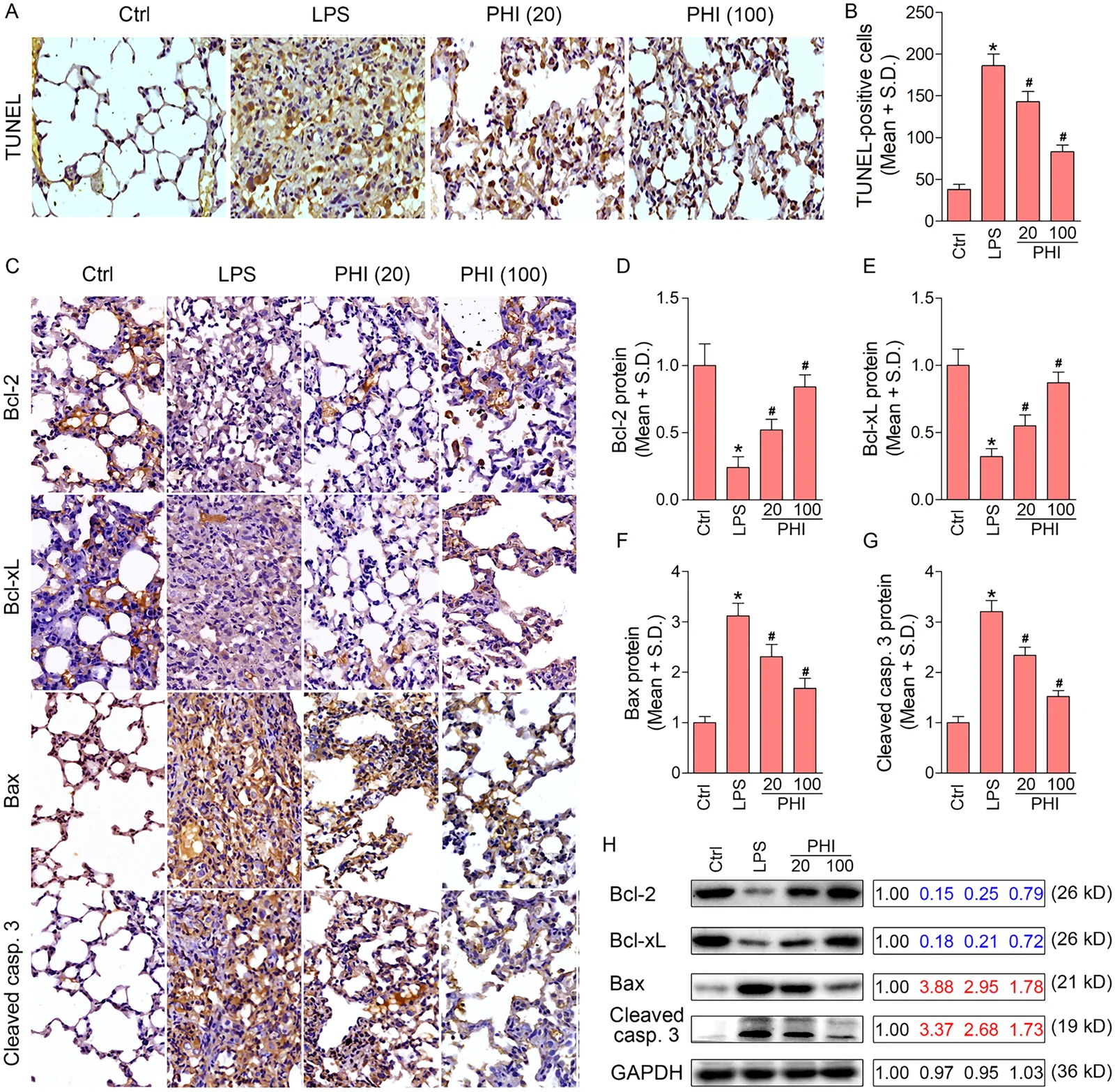
PHI suppresses LPS-induced apoptosis in lung tissue. Apoptosis in lung tissue was assessed by TUNEL staining (**A**,**B**). Apoptosis‑related proteins (Bcl‑2, Bcl‑xL, Bax, cleaved caspase‑3) were localized by immunohistochemistry (**C**–**G**) and quantified by western blotting in lung tissues (**H**). Data are presented as mean ± S.D. (*n* = 3). *P < 0.05 vs. control; ^#^*P* < 0.05 vs. LPS group. Original magnification: 40× (**A**,**C**). Full-length, uncropped blots are presented in Supplementary Figure S2.

**Fig. 7:**
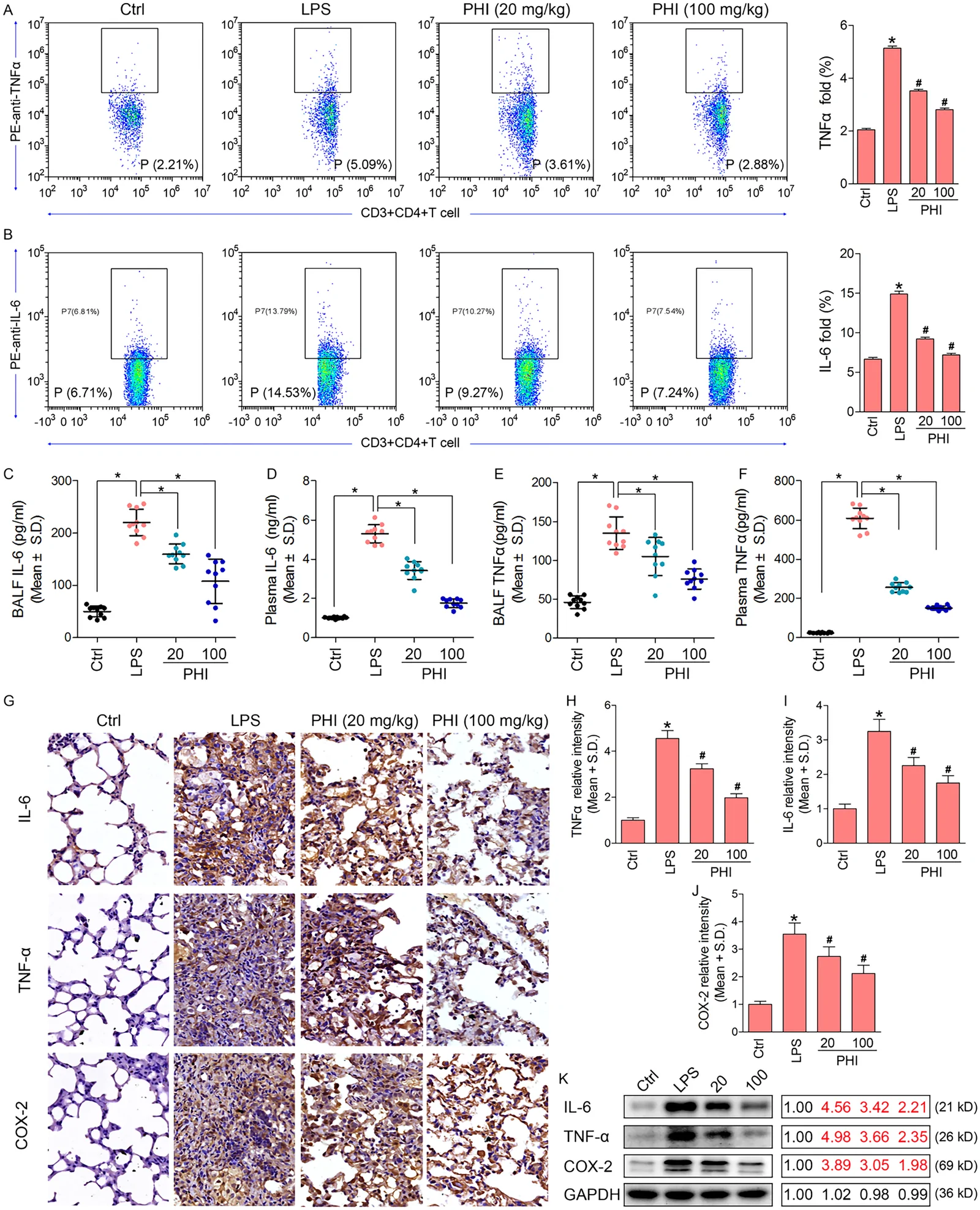
PHI reduces systemic and pulmonary inflammation induced by LPS in vivo. Levels of TNF‑α and IL‑6 were analyzed by flow cytometry in BALF immune cells (**A**,**B**) and by ELISA in BALF (**C**,**E**) and plasma (**D**,**F**). The protein levels of IL-6, TNFα, and COX-2 in lung tissues were visualized using IHC (**G**–**J**) and confirmed by western blot (**K**). Data are presented as mean ± S.D. (*n* = 3). *P < 0.05 vs. control; #*P* < 0.05 vs. LPS group. Original magnification: 40× (**G**). Full-length, uncropped blots are presented in Supplementary Figure S2.

Building on these findings, we evaluated apoptosis and inflammation in lung tissue. TUNEL assays demonstrated that PHI administration markedly attenuated LPS‑mediated apoptotic injury in the lungs (Fig. 6A,B). Consistently, Western blot and immunohistochemical (IHC) analyses confirmed that PHI concentration‑dependently downregulated pro‑apoptotic markers (Bax, cleaved caspase‑3) and upregulated anti‑apoptotic proteins (Bcl‑2, Bcl‑xL) (Fig. 6C–H). Given the critical role of acute inflammation in lung injury progression^2,3^, we also assessed inflammatory mediators. Compared to LPS stimulation alone, PHI markedly suppressed inflammatory responses, as shown by reduced levels of TNF‑α and IL‑6 in bronchoalveolar lavage fluid and serum (Fig. 7A–F), and by decreased expression of TNF‑α, IL‑6, and COX‑2 in lung tissues (Figs. 7G-K). Together, these results illustrated that PHI exerts integrated anti‑apoptotic and anti‑inflammatory effects in vivo.

In summary, PHI alleviates LPS‑induced acute lung injury through a PXR‑dependent mechanism that concurrently suppresses NF‑κB‑driven inflammation, enhances antioxidant defenses, and inhibits apoptosis.

## Discussion

This study establishes phillygenin (PHI) as a novel agonist of the pregnane X receptor (PXR) and defines a previously unrecognized PHI-PXR-NF-κB protective axis in LPS-induced acute lung injury (ALI). Building upon prior descriptive studies, we provide direct evidence for PHI-PXR binding, functional activation, and NF-κB suppression via competitive RXRα binding. PHI confers multi-faceted protection through this coactivator-level, PXR-dependent mechanism that inhibits NF-κB-driven inflammation while upregulating antioxidant and anti-apoptotic programs. Collectively, these results identified PHI-dependent PXR activation as a protective mechanism against ALI and underscore the translational potential of targeting PXR-NF-κB axis for the development of novel therapeutics.

Mechanistic Insights into PXR-RXRα-NF-κB Axis Regulation. A crucial insight pertains to competition for the coactivator RXRα. Previous work has shown that PXR activation can suppress NF-κB through transcriptional repression^41,42^. Here, we reveal a more direct physical mechanism: PHI-activated PXR competes with NF-κB p65 for RXRα binding (Fig. 2). This model aligns with the established role of RXRα as a promiscuous heterodimerization partner^43,44^, but importantly extends this concept to competitive interactions with a major inflammatory transcription factor. Similar competitive mechanisms have been proposed for other nuclear receptor pathways^41^. To our knowledge, this represents the first demonstration in the context of PXR-mediated lung protection. The concept of RXRα serving as a functional bridge between nuclear receptors and inflammatory pathways^45^ offers a fresh perspective on how PXR may fine-tune inflammatory responses at the cofactor level.

Pharmacological Advancement of Phillygenin. Our results greatly enhance the pharmacological understanding of PHI. Previous studies have established its general anti-inflammatory properties^27,28^. Additionally, certain phytochemicals have been reported to act as PXR ligands^30^. Distinct from these, we provide direct evidence that PHI operates through a coactivator-level competition that redirects RXRα binding from NF-κB p65 to PXR—a mechanism previously unrecognized in ALI pharmacotherapy. This specific target identification represents an important step from phenomenological observation to mechanistic understanding.

Expanding the Cytoprotective Repertoire of PXR. The protective benefits of PXR activation extend beyond NF-κB inhibition. Although PXR’s established role in xenobiotic metabolism is well known^18,22^, our findings demonstrate its ability to enhance the expression of crucial antioxidant enzymes (GPX-2/7) and anti-apoptotic proteins (Bcl-2/Bcl-xL) (Fig. 3). This coordinated action simultaneously counteracts two interconnected pathological features of ALI—oxidative stress and apoptosis^46^—suggesting that PXR activation may offer a more integrated therapeutic approach compared with targeting individual pathways. These findings expand the known cytoprotective functions of PXR beyond its metabolic roles^18,42^, positioning it as a multifaceted regulator of cellular homeostasis in inflammatory settings. Although the development of selective PXR antagonists and agonists has made considerable progress, well-validated tool compounds suitable for in vivo use remain scarce, which constitutes a technical bottleneck in this field.

Target Validation and Therapeutic Candidacy of PHI. The essential role of PXR was definitively demonstrated through loss-of-function experiments. As shown in Fig. 4, PXR knockdown completely abolished the protective effects of PHI, confirming PXR as the indispensable mediator of PHI action. This finding distinguishes PHI from compounds acting through pleiotropic or off-target mechanisms and underscores the on-target specificity of its pharmacological effects. These findings strengthen the candidacy of PHI as a promising lead compound and highlight PXR as a key node for therapeutic intervention.

Translational Concordance and Therapeutic Feasibility. The translational relevance of our findings is supported by concordance between in vitro and in vivo results. In LPS-challenged mice, PHI administration recapitulated the cellular findings, significantly ameliorating lung injury, reducing inflammatory infiltrates, and decreasing apoptosis (Fig. 5, 6 and 7). This dose-dependent protection across experimental systems demonstrates the pharmacological feasibility of modulating the PHI-PXR-NF-κB axis and supports PHI's potential for further clinical translation.

Study Limitations. First, our mechanistic findings are primarily based on an LPS-induced ALI model. This model, though standard^47,48^, represents only one form of a clinically heterogeneous condition^49^. Whether the PHI-PXR-NF-κB axis is protective against ALI induced by viral infection, sterile injury, or ventilator damage remains unconfirmed. Second, our study focuses on epithelial pathways. This clarifies epithelial-centric mechanisms but does not extend to macrophages, neutrophils, endothelial cells, and fibroblasts^50^. Therefore, while our results are valid within this experimental system, further validation in more complex, multi-cellular contexts is essential to fully assess the therapeutic potential and off-target effects.

Future Directions. Future studies should validate the PHI-PXR axis in diverse ALI models and delineate its cell-specific mechanisms. Subsequent work should also advance lead optimization to assess its therapeutic potential against post-injury lung fibrosis and in broader inflammatory contexts.

Collectively, this study delineates a novel ALI-protective mechanism mediated by phillygenin (PHI) through direct PXR agonism. It reveals that PHI competitively inhibits NF-κB signaling while coordinately enhancing cellular antioxidant and anti-apoptotic defenses. These results greatly enhance the pharmacological profiling of PHI. Furthermore, they validate PXR as a strategic therapeutic target and provide a mechanistic foundation for developing novel treatments for ALI/ARDS.

## Methods

### Ethics statement

Animals were procured from Vital River Laboratory Animal Technology (Beijing, China). This study was conducted in compliance with the ARRIVE guidelines 2.0 and the National Institutes of Health Guide for the Care and Use of Laboratory Animals. The experimental protocol was reviewed and approved by the Ethics Committee of Yantai Yuhuangding Hospital (approval number: 2023 − 398, dated November 29, 2023).

### Animal treatment

Male C57BL/6 mice (*n* = 40, 8–10 weeks, 23–28 g) were housed under standard conditions with a 12-hour light/dark cycle and free access to food and water. After 7 days of acclimatization, mice were randomly divided into four groups (*n* = 10): control (vehicle), LPS (LPS + vehicle), PHI low-dose: LPS + PHI (10 mg/kg, cumulative 20 mg/kg), and PHI high-dose: LPS + PHI (50 mg/kg, cumulative 100 mg/kg). PHI or vehicle was administered intragastrically once daily for two consecutive days. Two hours after the final administration, all groups, excluding the control group, received an intraperitoneal injection of LPS (20 mg/kg, E. coli O111:B4). At 8 h post-LPS, bronchoalveolar lavage fluid (BALF), blood samples, and lung tissue were collected from all the animals. Three animals per group were randomly selected for molecular and histological analyses.

### Reagents, antibodies, and kits

The following reagents were used: phillygenin (PHI, ≥ 98.0%, HY‑N0483, MedChemExpress), a lignan constituent of *Forsythia suspensa*; lipopolysaccharide (LPS, ≥ 98%, E. coli O111:B4, L8880, Solarbio); pregnenolone 16α-carbonitrile (PCN, ≥ 97%, P0543, Sigma-Aldrich); rifampicin (RIF, ≥97%, R3501, Sigma‑Aldrich); and Gomisin C (≥ 98%, HY-N0799, MedChemExpress).

Antibodies included: anti‑PXR (ab118336, Abcam); anti‑NF‑κB p65 (ab288751, Abcam); anti‑phospho‑NF‑κB p65 (Ser536, ab76302, Abcam); anti‑RXRα (ab125001, Abcam); anti‑GPX-2 (ab140130, Abcam); anti‑GPX-7 (ab96257, Abcam); anti‑Bcl‑2 (ab182858, Abcam); anti‑Bcl‑xL (ab32370, Abcam); anti‑IL‑6 (ab233706, Abcam); anti‑TNF‑α (ab215188, Abcam); anti‑iNOS (ab283655, Abcam); anti‑COX‑2 (ab283574, Abcam); anti‑Bax (ab182734, Abcam); anti‑GAPDH (ab9484, Abcam); anti‑Lamin B1 (ab194109, Abcam); anti‑β‑actin (HC201‑01, TransGen); and anti‑cleaved caspase‑3 (9661, CST). All reagents were purchased from commercial suppliers unless otherwise noted.

Commercial kits were used to measure glutathione peroxidase (GPx) activity (BC1195), reduced glutathione (GSH, BC1170), oxidized glutathione (GSSG, BC1185), nitric oxide (NO, BC1475), and reactive oxygen species (ROS, CA1410) (all Solarbio).

### Cell culture

Human bronchial epithelial cell lines, BEAS-2B (ATCC CRL-9609) and 16HBE (ATCC CRL-2741), were obtained from the China Center for Type Culture Collection (Wuhan, China). Cells were maintained according to established protocols. Briefly, they were cultured in complete RPMI 1640 medium (Gibco, USA) supplemented with 10% fetal bovine serum (FBS) and 1% penicillin‑streptomycin at 37 °C with 5% CO₂.

### Cell viability and apoptosis assays

For cell viability, cells were plated in 96-well plates (4,000 cells/well) and exposed to PHI alone (concentrations ranging from log₂[PHI, μM] = −8 to 8; 3.906 nM to 256 μM) for 24 h, or with LPS (1.0 μg/mL) for 12, 24, and 48 h, or pre-treated with LPS for 6 h followed by PHI treatment for 24 h. Viability was assessed using the Cell Counting Kit-8 (CCK-8) (Dojindo, Kumamoto, Japan) according to the manufacturer’s instructions.

Apoptosis was analyzed by flow cytometry using an Annexin V‑FITC/PI detection kit (BD Biosciences, USA). Cells were stained according to the manufacturer’s protocol and analyzed on a FACSCalibur flow cytometer (BD Biosciences, USA).

### Ligand fishing assay

PHI-PXR binding was assessed by hollow fiber-based ligand fishing (HFLF). Recombinant human PXR protein (SPR2158, Sigma-Aldrich, China) and purified mouse PXR protein (a generous gift from Professor Jie Lei, Shandong Medical and Pharmaceutical University) were employed for species-specific binding analysis. Hollow fiber membranes (GE Healthcare; inner diameter: 500 μm, pore size: 0.2 μm) were pre-treated using acetone, methanol, and deionized water (ddH₂O). PXR protein was then injected into the fiber lumen. The filled fibers were heat‑sealed, immersed in PHI solution (0–100 µg/mL), and incubated at 37°C for 30 min in an ultrasonic bath. After incubation, the hollow fiber membranes were rinsed three times with phosphate‑buffered saline (PBS). The retained complexes were then eluted with methanol and analyzed by liquid chromatography-mass spectrometry (LC‑MS). PCN and RIF were used as positive controls.

### Transient transfection and luciferase assays

16HBE and BEAS-2B cells were co-transfected with pGL3-CYP3A4-promoter (Addgene, USA) and pcDNA3.1-PXR (human and mouse constructs) using Lipofectamine 3000 (Invitrogen, USA). At 48 h post-transfection, cells were exposed to either PHI (4, 16 nM) or PCN (4, 16 µM, PXR agonist positive control) for 24 h. Subsequently, cells were co-treated for an extra 24 h with LPS (1.0 μg/mL) and/or Gomisin C (2.5 μg/mL, NF-κB inhibitor). Luciferase activity was measured using the Dual‑Luciferase Reporter Assay System (Promega, USA), with firefly luciferase signals normalized to Renilla luciferase activity. Each sample was tested in triplicate.

### Histological analysis

Mouse lung pathology was evaluated by hematoxylin‑eosin (H&E) and Masson staining. Briefly, tissue specimens were fixed overnight in 4% paraformaldehyde, dehydrated, cleared, paraffin‑embedded, and sectioned at 2 μm. For H&E staining, the tissue sections were stained with H&E dyes and then sealed with a mounting medium for subsequent microscopic observation. For Masson staining, sections were sequentially stained with hematoxylin, Ponceau S, and phosphomolybdic acid, followed by aniline blue counterstaining. Subsequently, each tissue sample was incubated in 1% acetic acid solution for 1 min and observed under a microscope. Lung tissue apoptosis was evaluated using TdT-mediated dUTP nick end labeling (TUNEL) staining, in accordance with the manufacturer’s instructions.

Protein expression in lung tissue was assessed by immunohistochemistry (IHC). Paraffin‑embedded sections were deparaffinized, rehydrated, subjected to antigen retrieval in citrate buffer (10 mM, pH 6.0), followed by blocking and incubation with primary antibodies, washed with PBS, and subsequently probed with HRP‑conjugated secondary antibodies.

### Immunofluorescence and western blotting

16HBE and BEAS-2B cells were placed into confocal dishes at 5 × 10^5^ cells/mL and maintained for 24 h. Cells were treated with LPS in the presence or absence of PHI for 24 h, fixed with 4% paraformaldehyde (20 min, RT), permeabilized with 0.1% Triton X‑100, and blocked with 5% goat serum. After overnight incubation with NF‑κB p65 antibody (1:100) at 4 °C, cells were washed three times with PBS and incubated with PE‑conjugated goat anti‑rabbit IgG (1:200) for 1 h at RT. Nuclei were counterstained with DAPI.

Protein levels were assayed by Western blotting (WB). After LPS treatment, 16HBE and BEAS‑2B cells were cultured either with or without PHI, and subsequently lysed in RIPA buffer containing PMSF. Protein concentrations were measured using a BCA kit (Solarbio). Equal protein amounts (20 µg/lane) were resolved via SDS‑PAGE, transferred to PVDF membranes, blocked with 5% non‑fat milk, and incubated with primary antibodies diluted as follows: anti-phospho-NF-κB p65 (1:1000), anti‑NF‑κB p65 (1:800), anti-Bcl-2 (1:1500), anti-IL-6 (1:1500), anti-TNF-α (1:1500), anti-Bax/Bcl-xL/cleaved caspase-3/COX‑2/iNOS (1:1000), anti-PXR/RXRα/GPX-2/GPX-7 (1:2000), anti‑β‑actin (1:6000), anti‑GAPDH (1:10000), and anti‑Lamin B1 (1:3000) for 2 h at RT. Membranes were subsequently incubated with HRP‑conjugated secondary antibodies for 1 h, washed with TBST, and visualized using an enhanced chemiluminescence system (Bio‑Rad).

Western blot analysis and protein identification. Primary antibodies were used against: PXR (~ 50 kDa), RXRα (~ 54 kDa), p65 (~ 65 kDa), GPx-2 (~ 22 kDa), GPx-7 (~ 19 kDa), cleaved caspase-3 (~ 17 kDa), Bax (~ 21 kDa), Bcl-2 (~ 26 kDa), iNOS (~ 130 kDa), COX-2 (~ 72 kDa), β-actin (~ 42 kDa), Lamin B1 (~ 66 kDa), and GAPDH (~ 36 kDa). Target proteins were assigned based on antibody-specific expected molecular weight (manufacturer datasheets, published validation), multi-housekeeping protein internal standards (β-actin, GAPDH, Lamin B1), and replicate consistency (*n* = 3). This marker-independent validation strategy provided unambiguous protein identification. Uncropped blot images are provided in Supplementary Figure S2.

### Cytokine measurement

Levels of IL‑6 and TNF‑α in BALF supernatant and serum were assessed using ELISA (BioLegend, USA) following the manufacturer’s instructions. Absorbance was read at 450 nm using a PowerWave‑X spectrophotometer (BioTek, USA). For intracellular cytokine detection in BALF immune cells, surface staining was performed with FITC‑conjugated anti‑CD3 and APC‑conjugated anti‑CD4 antibodies. Cells were then fixed and permeabilized, followed by intracellular staining with PE‑conjugated anti‑IL‑6 or anti‑TNF‑α antibodies (BioLegend, USA). The absorbance was read at 450 nm using the Power Wave-X spectrophotometer (BioTek, USA). Flow cytometric analysis was performed on the CellQuest software program (BD Biosciences, USA) for data acquisition and analysis.

### ROS level detection

For ROS detection, 16HBE and BEAS-2B cells underwent transient transfection for PXR overexpression followed by LPS exposure. The cells were stained with DCFH-DA (1 mM, 30 min) and washed with PBS. ROS levels were assessed through flow cytometry using DCFH‑DA staining, and data were analyzed using FlowJo software.

### Nitric oxide, glutathione, and the oxidized glutathione analysis

Levels of nitric oxide (NO, BC1475), reduced glutathione (GSH, BC1170), and oxidized glutathione (GSSG, BC1185) in 16HBE and BEAS-2B cells were determined using corresponding commercial kits (Solarbio, China) according to the manufacturer’s instructions.

### Statistical analysis

Data are expressed as mean ± SD. Statistical analysis was conducted using SPSS 26.0 (IBM, USA). One‑way ANOVA was applied to normally distributed data with homogeneous variance; otherwise, non‑parametric tests were utilized. Differences were considered significant at *P* < 0.05.

## Supplementary Information

Below is the link to the electronic supplementary material.

## Data Availability

All data supporting the findings of this study are available within the paper and its Supplementary Information.

## Abbreviations

ALI: Acute lung injury
ARDS: Acute respiratory distress syndrome
PHI: Phillygenin
LPS: Lipopolysaccharide
PXR: Pregnane X receptor
NF-κB: Nuclear factor kappa-B
RIF: Rifampicin
RXRα: Retinoid X receptor alpha
iNOS: Inducible nitric oxide synthase
AEC: Alveolar epithelial cell
ROS: Reactive oxygen species
GPX: Glutathione Peroxidase
GSH: Reduced Glutathione
GSSG: Oxidized Glutathione
NO: Nitric Oxide
PBS: Phosphate-buffered saline
LC-MS: Liquid chromatography-mass spectrometry
IHC: Immunohistochemical staining
BALF: Bronchoalveolar Lavage Fluid
HBE: Human bronchial epithelial cell line
