# Supplementary material for: Phillygenin protects against lipopolysaccharide-induced acute lung injury by acting as a PXR agonist via NF-κB inhibition
Source: Sci Rep. 2026 Aug 29;16:27535. doi: 10.1038/s41598-026-66771-z (PMC13538703; doi:10.1038/s41598-026-66771-z)
Supplement: Supplementary file 1 [file 41598_2026_66771_MOESM1_ESM.pdf]

Figure S1

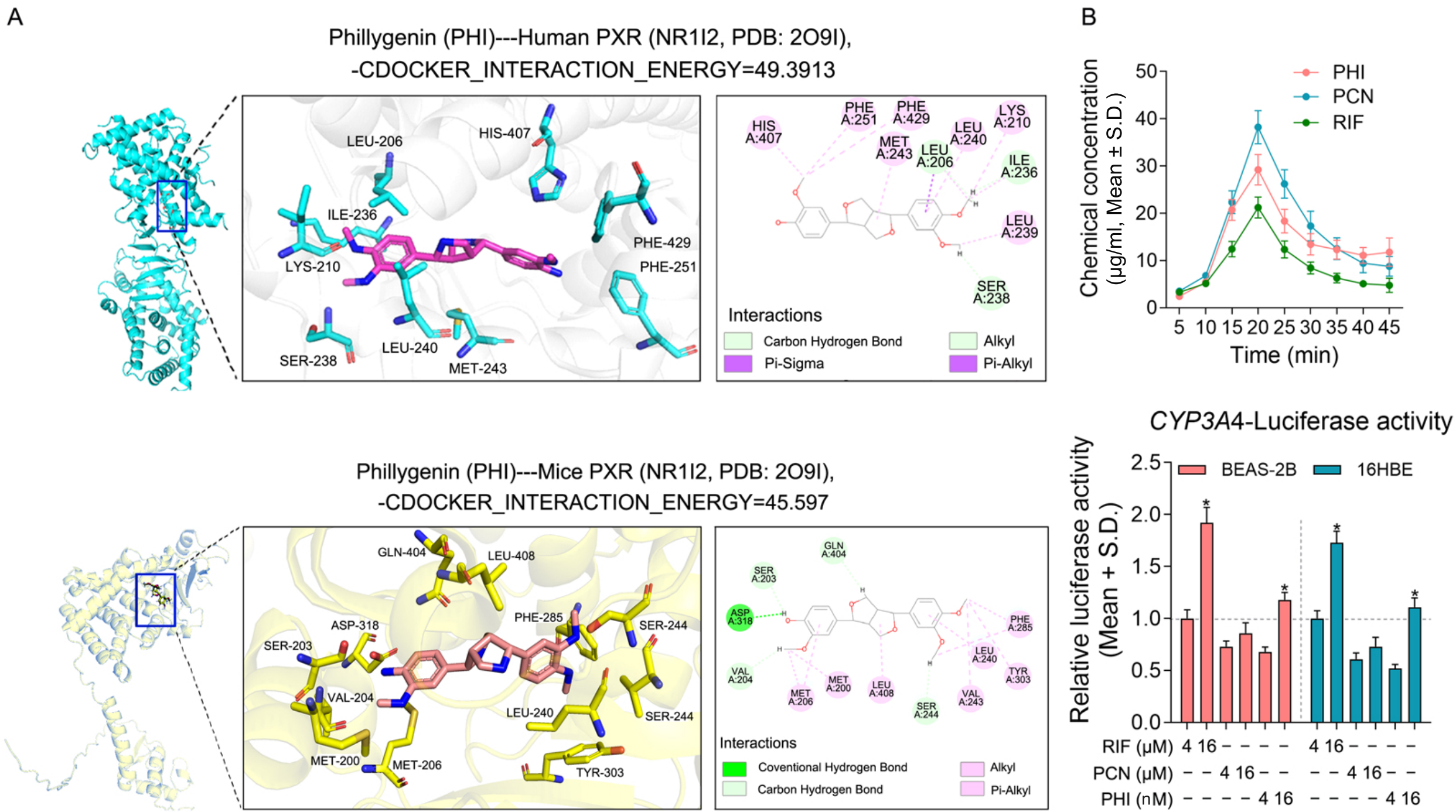

A
Phillygenin (PHI)---Human PXR (NR1I2, PDB: 2O9I),
-CDOCKER_INTERACTION_ENERGY=49.3913
LEU-206
HIS-407
ILE-236
LYS-210
PHE-429
PHE-251
SER-238
LEU-240
MET-243
HIS A:407
PHE A:251
PHE A:429
MET A:243
LEU A:206
LEU A:240
LYS A:210
ILE A:236
LEU A:239
SER A:238
Interactions
Carbon Hydrogen Bond
Alkyl
Pi-Sigma
Pi-Alkyl
Phillygenin (PHI)---Mice PXR (NR1I2, PDB: 2O9I),
-CDOCKER_INTERACTION_ENERGY=45.597
GLN-404
LEU-408
ASP-318
PHE-285
SER-244
SER-203
VAL-204
LEU-240
SER-244
MET-200
MET-206
TYR-303
GLN A:404
SER A:203
ASP A:318
VAL A:204
MET A:206
MET A:200
LEU A:408
SER A:244
VAL A:243
LEU A:240
PHE A:285
TYR A:303
Interactions
Coventional Hydrogen Bond
Carbon Hydrogen Bond
Alkyl
Pi-Alkyl
B
Chemical concentration (μg/ml, Mean ± S.D.)
Time (min)
PHI
PCN
RIF
CYP3A4-Luciferase activity
Relative luciferase activity (Mean + S.D.)
BEAS-2B
16HBE
RIF (μM) 4 16 – – – – 4 16 – – – –
PCN (μM) – – 4 16 – – – – 4 16 – –
PHI (nM) – – – – 4 16 – – – – 4 16

Figure S2

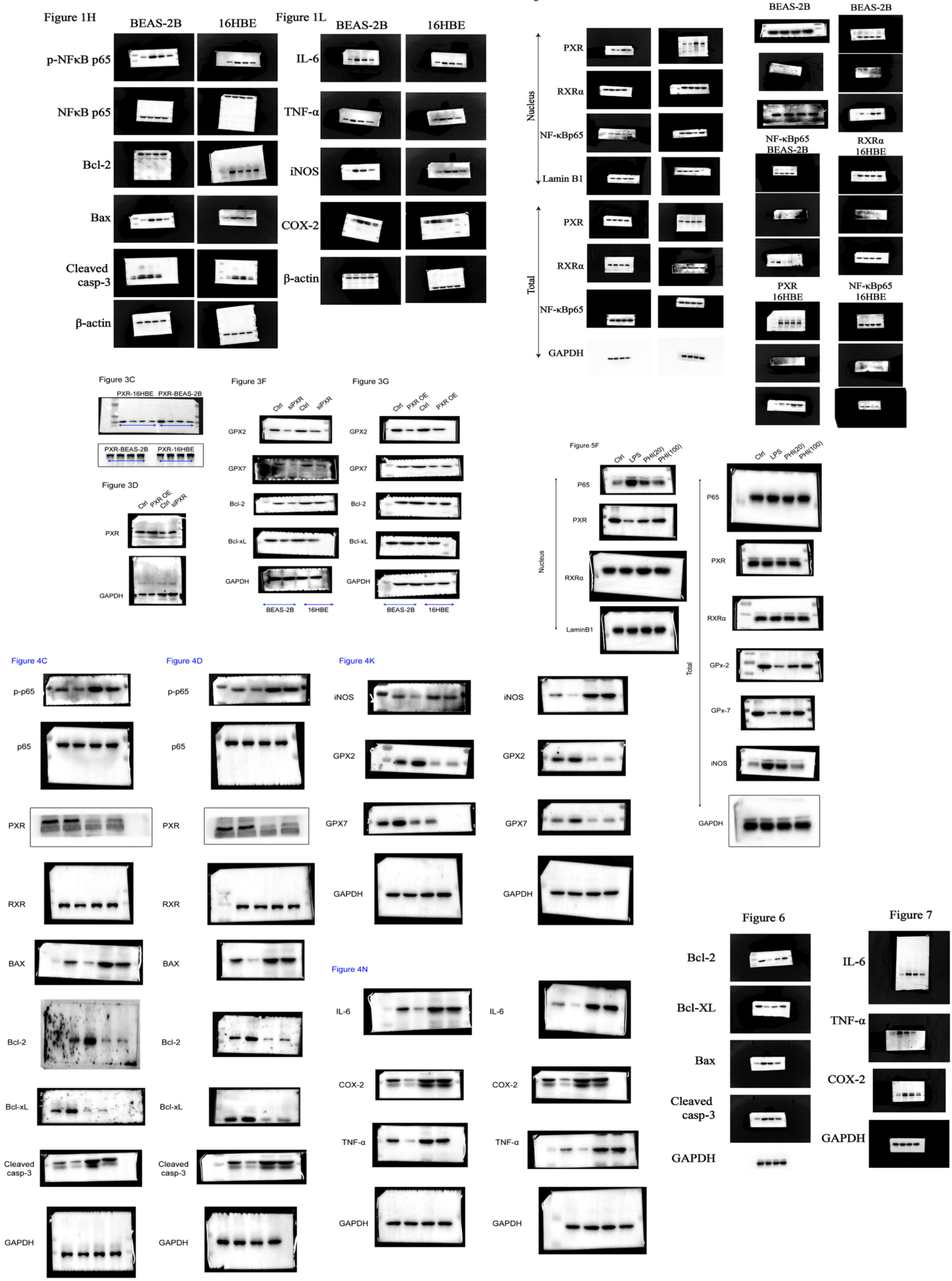
Figure 1H
BEAS-2B
16HBE
p-NFκB p65
NFκB p65
Bcl-2
Bax
Cleaved casp-3
β-actin
Figure 1L
BEAS-2B
16HBE
IL-6
TNF-α
iNOS
COX-2
β-actin
Figure 2F
Nucleus
Total
PXR
RXRα
NF-κBp65
Lamin B1
PXR
RXRα
NF-κBp65
GAPDH
Figure 2H
RXRα BEAS-2B
PXR BEAS-2B
NF-κBp65 BEAS-2B
RXRα 16HBE
PXR 16HBE
NF-κBp65 16HBE
Figure 3C
PXR-16HBE
PXR-BEAS-2B
PXR-BEAS-2B
PXR-16HBE
Figure 3D
Ctrl
PXR OE
Ctrl
siPXR
PXR
GAPDH
Figure 3F
Ctrl
siPXR
Ctrl
siPXR
GPX2
GPX7
Bcl-2
Bcl-xL
GAPDH
BEAS-2B
16HBE
Figure 3G
Ctrl
PXR OE
Ctrl
PXR OE
GPX2
GPX7
Bcl-2
Bcl-xL
GAPDH
BEAS-2B
16HBE
Figure 5F
Ctrl
LPS
PHI(20)
PHI(100)
Nucleus
P65
PXR
RXRα
LaminB1
Ctrl
LPS
PHI(20)
PHI(100)
Total
P65
PXR
RXRα
GPx-2
GPx-7
iNOS
GAPDH
Figure 4C
p-p65
p65
PXR
RXR
BAX
Bcl-2
Bcl-xL
Cleaved casp-3
GAPDH
Figure 4D
p-p65
p65
PXR
RXR
BAX
Bcl-2
Bcl-xL
Cleaved casp-3
GAPDH
Figure 4K
iNOS
GPX2
GPX7
GAPDH
iNOS
GPX2
GPX7
GAPDH
Figure 4N
IL-6
COX-2
TNF-α
GAPDH
IL-6
COX-2
TNF-α
GAPDH
Figure 6
Bcl-2
Bcl-XL
Bax
Cleaved casp-3
GAPDH
Figure 7
IL-6
TNF-α
COX-2
GAPDH
